# Clinicopathological Differences between Right and Left Colorectal Cancer by Sex

**DOI:** 10.3390/jcm13102810

**Published:** 2024-05-10

**Authors:** Hannah Ra, Soyeon Jeong, Hannah Lee, Jun-Won Chung, Kyoung Oh Kim, Won-Suk Lee, Jisup Kim, Kwang An Kwon, Jung Ho Kim

**Affiliations:** 1 Department of Internal Medicine, Gachon University Gil Medical Center, Gachon University College of Medicine, Incheon 21565, Republic of Korea; hannahra@gilhospital.com (H.R.); hannahlee@gilhospital.com (H.L.); drgreen@gilhospital.com (J.-W.C.); kkoimge@gilhospital.com (K.O.K.); toptom@gilhospital.com (K.A.K.); 2 Gachon Biomedical Convergence Institute, Gachon University Gil Medical Center, College of Medicine, Incheon 21565, Republic of Korea; jensyj85@gmail.com; 3 Gachon Medical Research Institute, Gachon University Gil Medical Center, Gachon University College of Medicine, Incheon 21565, Republic of Korea; 4 Department of Surgery, Gachon University Gil Medical Center, Gachon University College of Medicine, Incheon 21565, Republic of Korea; lws@gilhospital.com; 5 Department of Pathology, Gachon University Gil Medical Center, Gachon University College of Medicine, Incheon 21565, Republic of Korea; jspath@gilhospital.com; 6 Department of Translational-Clinical Medicine, Gachon Advanced Institute for Health Sciences and Technology (GAIHST), Gachon University, Incheon 21999, Republic of Korea

**Keywords:** colorectal cancer, location, sex, neoplasm

## Abstract

**Background**: Until now, studies on colorectal cancer (CRC) have focused on clinicopathological characteristics based on location without considering sex differences. However, as men and women have fundamentally different physiological characteristics, research results in the clinical field are limited. We aimed to elucidate the differences in the clinicopathological characteristics between right-sided CRC (RCC) and left-sided CRC (LCC) according to sex. **Methods**: We classified 1492 South Korean patients with no history of colon surgery between July 2005 and June 2015 based on tumor location and sex. For these patients, differences in the clinical characteristics according to sex were compared using univariate and multivariate analyses. **Results**: Of the 1269 patients, 951 (74.9%) had LCC, and 318 (25.1%) had RCC, making LCC approximately three times more common than RCC. When sex was not taken into account, patients with RCC had significantly higher rates of anemia and undifferentiated cancers than the rates in those with LCC. Even considering sex, anemia and undifferentiated cancer were more prevalent in RCC than in LCC in both men and women. In contrast, age over 65 years and abnormal white blood cell count differed between RCC and LCC only in women. **Conclusions**: The clinicopathologic characteristics of CRC vary according to the location and sex. Therefore, sex must be considered as a fundamental characteristic of personalized treatment.

## 1. Introduction

Colorectal cancer (CRC) ranks third in cancer incidence worldwide, followed by cancer-related mortality [1]. To date, research has mainly focused on the clinicopathological characteristics of the disease according to tumor site, including right-sided CRC (RCC) and left-sided CRC (LCC) [2,3]. This is because the molecular biological mechanisms, symptoms, and signs differ depending on the location of CRC [2,3]. An intriguing fact is that the proximal and distal colon originates from distinct embryonic sources. Specifically, the cecum, ascending colon, and the proximal two-thirds of the transverse colon are derived from the embryonic midgut. At the same time, the hindgut gives rise to the distal third of the transverse colon, descending colon, sigmoid colon, and rectum. This variance in embryological origin plays a crucial role in various colon sections’ diverse features and roles [4]. The proximal and distal colon have different blood supplies, with the right colon supplied by the superior mesenteric artery and the left colon by the inferior mesenteric artery [5].

Moreover, RCC and LCC have different initial symptoms. RCC presents with anemia and weight loss, while LCC presents with changes in bowel movement and hematochezia [6]. The prognosis for RCC remains poor despite improvements observed in the prognosis for LCC since 1980 [7]. The distinct features of RCC and LCC have received attention in recent years owing to the differences in prognosis and clinical response to chemotherapy [8,9].

The age-standardized (AS) incidence rate of CRC is approximately 1.3–1.5 times higher in men than in women. The AS mortality rate is approximately 1.4–1.6 times higher in men than in women [1], indicating sex-dependent differences in disease incidence and mortality. Historically, women were excluded as participants in non-reproductive clinical research. As a result, data from investigations focused on men were extrapolated to apply to women [10]. However, differences in biology may potentially impact disease development and treatment efficacy. Additionally, considering that men and women have fundamentally distinct physiologies [11], carcinogenesis, risk factors, response to chemotherapy, and prognosis may differ between the sexes. The influence of sex on CRC mechanisms has not been sufficiently investigated, and the classification of LCC/RCC based on sex differences is expected to yield significant results.

Determining how sex affects CRC prognosis can provide new insights into the risk factors, early diagnosis, therapy, and strategies to improve survival, allowing for personalized treatment. This study aimed to reveal the differences in the clinicopathological characteristics of CRC based on anatomic location and sex.

## 2. Materials and Methods

### 2.1. Patients

**Figure 1:**
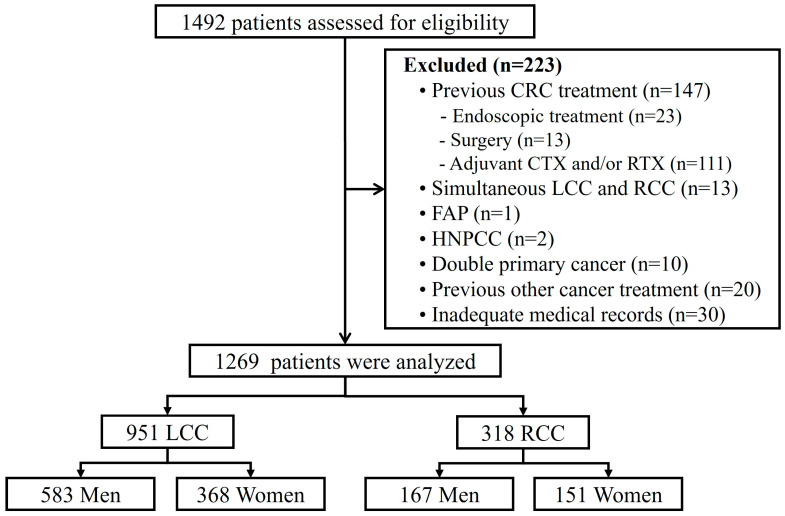
Flow chart of the study. CRC: colorectal cancer, CTX: chemotherapy, FAP: familial adenomatous polyposis, HNPCC: hereditary nonpolyposis colorectal cancer, LCC: left-sided colorectal cancer, RCC: right-sided colorectal cancer; RTX: radiation therapy.

We retrospectively reviewed the medical records of patients diagnosed with CRC who were surgically treated between July 2005 and June 2015 at the Gachon University Gil Medical Center. We reviewed the data of 1492 patients diagnosed with CRC for the final pathological diagnosis. The following patients were excluded from the analysis (Figure 1). Approximately, 147 patients previously treated for CRC were excluded from the study. Of these patients, 23 underwent endoscopic resection only, 13 underwent surgery, and 111 received chemotherapy and/or radiotherapy. 13 patients with simultaneous LCC and RCC, 1 with familial adenomatous polyposis, and 2 with hereditary nonpolyposis were also excluded. At the time of CRC diagnosis, 10 patients with cancers in other organs and 20 patients who had undergone abdominal surgery, systemic chemotherapy, or radiation therapy while receiving other cancer treatments in the past were also excluded. Furthermore, 30 patients with incomplete pathological results or staging information were also excluded. Finally, we analyzed data from 1269 patients who underwent initial diagnosis and surgical resection. The Institutional Review Board of the Gachon University Gil Medical Center approved this study (IRB No. GBIRB2018-407).

### 2.2. Variables

Family history (FHX) was defined as the occurrence of CRC in a first-degree relative. Smoking status was determined by past or present smoking. Anemia was defined as hemoglobin levels below 13 g/dL in men and 12 g/dL in women. Abnormal white blood cell (WBC) count is generally referred to as leukocytosis (over 10,000 cells per μL) or leukopenia (below 4000 cells per μL). An abnormal carcinoembryonic antigen (CEA) level was defined as 5 ng/mL or more, and the derived neutrophil-to-lymphocyte ratio (dNLR), a clinical hematological biomarker, was calculated using the following formula: dNLR = absolute neutrophil count (ANC)/(WBC count − ANC) [12]. CRC was classified as differentiated or undifferentiated based on the following pathological characteristics: (1) differentiated: well-differentiated (WD) and moderately differentiated (MD) tumors, (2) undifferentiated: poorly differentiated (PD) tumors, and tumors with signet ring cells and mucinous carcinomas.

The location of its primary tumor characterizes CRC. We defined RCC as cancer of the cecum and ascending colon up to the transverse colon; meanwhile, we defined LCC as the cancer of the splenic flexure and regions distal to the splenic flexure, including the rectum [8]. Finally, patients with CRC were divided into LCC and RCC groups according to sex.

### 2.3. Statistical Analysis

Continuous variables are expressed as the mean with standard deviation, whereas categorical variables are presented as absolute numbers and percentages. SPSS (version 22.0; SPSS Inc., Chicago, IL, USA) was used for the statistical analyses. In univariate analysis, categorical data were analyzed using Pearson’s chi-square or Fisher’s exact test. Multivariate analysis was performed using binary logistic regression for significant factors in univariate analysis, and the results were expressed as odds ratios (OR) with 95% confidence intervals (CI). *p* < 0.05 was considered to indicate statistical significance.

## 3. Results

### 3.1. Baseline Characteristics of CRC

The baseline characteristics of the 1269 patients are summarized in Table 1. The mean age was 64.2 ± 11.8 years. In our cohort, 750 (59.1%) and 519 (40.9%) patients were men and women, respectively. Diabetes mellitus (DM) was diagnosed in 19.9% of the patients. LCC was identified in 74.9% of the patients, and RCC was diagnosed in 25.1% of the patients; thus, LCC was approximately three times more common than RCC. The most common histological grade was MD 87.6%, and most patients were diagnosed with pathological tumor, node, and metastasis (TNM) stage II (32.3%) or III (32.9%).

### 3.2. Differences between RCC and LCC without Considering Sex

Table 2 displays the results of comparing clinicopathological differences according to the CRC location. In univariate analysis, among 951 patients with LCC, 583 (61.3%) were men and 368 (38.7%) were women. Among the 318 patients with RCC, 167 (52.5%) were men and 151 (47.5%) were women. Additionally, smoking (*p* = 0.038), anemia (*p* < 0.001), and differentiated status (*p* < 0.001) were statistically significantly different depending on tumor location. In the multivariate analysis, the rates of anemia (OR: 2.338; 95% CI: 1.779–3.074; *p* < 0.001) and undifferentiated cancers (OR: 3.223; 95% CI: 1.900–5.467; *p* < 0.001) were significantly higher in patients with RCC than in those with LCC. No significant relationships were observed between tumor location and the following clinicopathological parameters: age, sex, smoking status, presence of DM, FHX, WBC status, CEA level, dNLR, presence of p53/epidermal growth factor receptor (EGFR) mutation, and TNM stage.

### 3.3. Sex-Specific Clinicopathological Differences between RCC and LCC

**Figure 2:**
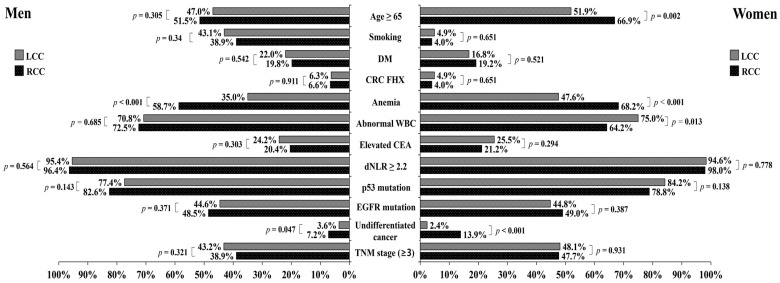
Univariate analysis of sex-specific difference in colon cancer characteristics. CEA: carcinoembryonic antigen; CRC: colorectal cancer; dNLR: derived neutrophil-to-lymphocyte ratio; DM: diabetes mellitus; EGFR: epidermal growth factors receptor; FHX: family history; LCC: left-sided colorectal cancer; RCC: right-sided colorectal cancer; TNM stage: tumor, node; metastasis staging system; WBC: white blood cell.

Next, we analyzed differences in the clinicopathological characteristics of LCC and RCC according to sex. Figure 2 presents the results of the analysis. In the univariate analysis, both men and women with RCC presented with a higher proportion of anemia (*p* < 0.001) and PD cancer (*p* = 0.047 and *p* < 0.001, respectively) than those with LCC. In contrast, women over 65 years (*p* = 0.002) were more frequently diagnosed with RCC than with LCC, and an abnormal WBC count (*p* = 0.013) was less common in RCC than in LCC.

In the multivariate analysis, we examined factors that were significant in the univariate analysis (Table 3). For both men and women patients, anemia (OR: 2.620; 95% CI: 1.841–3.727; *p* < 0.001, OR: 1.976; 95% CI: 1.301–3.000; *p* = 0.001, respectively) was an independent factor that demonstrated a statistically significant difference in RCC. The incidence of undifferentiated cancer tended to be high in men with RCC and significantly higher in women with RCC than in men. Interestingly, in women with RCC compared to LCC, age (over 65 years, OR: 1.587; 95% CI: 1.044–2.414; *p* = 0.031) and abnormal WBC count (OR: 0.587; 95% CI: 0.383–0.900; *p* = 0.014) were significantly different factors, whereas these differences were not observed in men.

## 4. Discussion

Although differences in tumor location have long been considered crucial in CRC research due to variations in occurrence, molecular pathogenesis, and prognosis based on tumor location [13], accumulating evidence suggests that clinicopathological characteristics, treatment response, and prognosis also differ according to sex [9]. Given the significance of recognizing sex as a fixed host factor, we attempted to reveal differences in clinicopathological characteristics by considering sex as a commonly assumed determinant of CRC occurrence. We confirmed that specific clinicopathological characteristics related to the CRC location differed according to sex.

The embryonic origins of RCC and LCC are distinct. Moreover, RCC develops in the midgut, whereas LCC develops in the hindgut. Several studies have reported the associations between carcinogenic risk factors, molecular pathways, and pathogenic mechanisms with CRC location [14,15]. Therefore, differences depending on the anatomical location of CRC are currently considered important. Similar to other studies that reported differences between RCC and LCC [16,17,18,19], the present analysis demonstrated that patients with RCC had significantly higher rates of anemia and undifferentiated cancer than the rates in those with LCC. Even when sex was included in the univariate and multivariate analyses, the results remained unchanged.

Independent of sex, anemia and undifferentiated cancer occurred more frequently in patients with RCC than in those with LCC, which may be attributable to differences in genetic instability. Chronic blood loss is the primary cause of anemia in CRC [20]. According to previous studies, iron-deficiency anemia (IDA) occurs more often in RCC than in LCC. Consequently, the more frequent occurrence of anemia in RCC may be due to IDA caused by prolonged low-grade loss of iron-rich blood close to the base of the RCC, leading to the gradual depletion of body iron stores [21,22]. Iron deficiency is essential in deoxyribonucleic acid damage by reducing Fe–S clusters, leading to genomic instability [23].

Undifferentiated cancers, including PD CRC, are closely related to microsatellite instability (MSI)-H [24,25], and MSI-H PD cancer is more frequently present in RCC than in LCC [26]. Furthermore, unlike LCC, RCC is characterized by frequent gene alterations such as those in *BRAF* and *KRAS*, which may contribute to a high frequency of MSI-H [14,27]. Therefore, the elevated risk of anemia and undifferentiated cancer in patients with RCC may be attributed to the location of CRC onset rather than sex.

A notable sex-related clinicopathological difference between RCC and LCC was the higher proportion of women aged 65 years or older diagnosed with RCC than with LCC. No significant differences were observed among men. Thus, age can be considered a significant independent risk factor in women with RCC. This may be related to the decline in ovarian function observed in postmenopausal women aged > 65 years. As ovarian function declines, an increase in the incidence of RCC and the abundance of gut microbial β-glucuronidase has been observed [28,29]. These changes in the gut microbial can cause an imbalance in the immune response and chronic inflammation, thereby promoting RCC development [30]. In other words, in patients with RCC who are over 65 years of age, this result may be triggered by a disruption in the balance between inflammation and immune responses via changes in the gut microbial due to decreased ovarian function.

We identified no statistical difference between RCC and LCC when abnormalities in the WBC count were classified by location only. When the sex of the patient was considered, a reduced incidence of abnormal WBC count was noted in women with RCC. This may be related to hormone sensitivity. Estrogen is generally known to suppress inflammation by inhibiting the expression of inflammatory cytokines and regulating inflammation-related signaling pathways [31]. Among the various forms of estrogen, estradiol has the strongest anti-inflammatory effect. After menopause, estradiol levels decrease while androgen levels increase [32,33]. However, the expression of sex hormone receptors differs between LCC and RCC in women over 60 years [34]. Our cohort included numerous postmenopausal women over the age of 65 years who may experience increased inflammatory responses due to hormonal changes. However, the abnormal WBC reduction in women with RCC (reduced ratio of patients with leukocytosis) is probably due to decreased sensitivity to sex hormones caused by differences in hormone receptor expression.

This study has some limitations, as it was a retrospective study conducted at a single university hospital; therefore, the detailed mechanism underlying sex differences cannot be fully determined. Despite this limitation, we discovered that the specific characteristics of CRC based on location varied according to sex. We improved the reliability of the data by surveying a large cohort of over 1200 patients with CRC. Additionally, unlike other studies that examined survival by simply comparing other factors according to sex differences [35,36], this study used univariate and multivariate analyses, considering various clinical factors, to obtain more precise and accurate data. These results will serve as a benchmark for future research on the customized diagnosis and treatment of CRC. Conducting sex-related research is one of the several factors that must be considered in personalized medicine.

## 5. Conclusions

We revealed diverse sex-associated patterns across CRC locations. Based on these findings, future research on personalized cancer therapy and prevention should account for variations based on tumor location and the influence of sex. Additional investigations on clinically effective sex-associated strategies as fixed factors in CRC diagnosis and treatment are required.

## Figures and Tables

**Table 1:** Demographic data of the patients with CRC.

	Total (*n* = 1269)
Age (years)	64.2 ± 11.8
Sex	
Men	750 (59.1%)
Women	519 (40.9%)
DM	252 (19.9%)
Smoking	340 (26.8%)
CRC FHX	72 (5.7%)
Laboratory findings	
Hemoglobin (g/dL)	12.4 ± 2.4
WBC count	12,222.1 ± 3976.0
CEA level	98.3 ± 380.9
dNLR	7.7 ± 5.0
Tumor location	
LCC	951 (74.9%)
RCC	318 (25.1%)
Tumor size (mm)	46.6 ± 22.7
Grade of differentiation	
WD	94 (7.4%)
MD	1112 (87.6%)
PD	63 (5.0%)
p53 mutation	1018 (80.2%)
EGFR mutation	580 (45.7%)
TNM stage	
Stage 0	25 (2.0%)
Stage I	268 (21.1%)
Stage II	410 (32.3%)
Stage III	418 (32.9%)
Stage IV	148 (11.7%)

CEA, carcinoembryonic antigen; CRC, colorectal cancer; dNLR, derived neutrophil-to-lymphocyte ratio; DM, diabetes mellitus; EGFR, epidermal growth factor receptor; FHX, family history; LCC, left-sided colorectal cancer; MD, moderately differentiated cancer; PD, poorly differentiated cancer; RCC, right-sided colorectal cancer; TNM stage, tumor-node-metastasis staging system; WBC, white blood cell; WD, well-differentiated cancer.

**Table 2:** Differences in the clinicopathologic characteristics between RCC and LCC.

Variables	Univariate Analysis			Multivariate Analysis	
	LCC (*n* = 951)	RCC (*n* = 318)	*p*	OR (95% CI)	*p*
Age ≥ 65	465 (48.9%)	187 (58.8%)	0.002	1.218 (0.923–1.606)	0.163
Men	583 (61.3%)	167 (52.5%)	0.006	0.8 (0.598–1.070)	0.133
Women	368 (38.7%)	151 (47.5%)	0.004	0.698 (0.540–0.902)	0.006
Smoking	269 (28.3%)	71 (22.3%)	0.038	0.99 (0.702–1.398)	0.956
DM	190 (20.0%)	62 (19.5%)	0.852	NA	
CRC FHX	55 (5.8%)	17 (5.3%)	0.77	NA	
Anemia	379 (39.9%)	201 (63.2%)	<0.001	2.338 (1.779–3.074)	<0.001
Abnormal WBC count	689 (72.5%)	218 (68.6%)	0.183	NA	
Elevated CEA level	235 (24.7%)	66 (20.8%)	0.151	NA	
dNLR (≥2.2)	918 (96.5%)	309 (97.2%)	0.581	NA	
p53 mutation	761 (80.0%)	257 (80.8%)	0.758	NA	
EGFR mutation	425 (44.7%)	155 (48.7%)	0.209	NA	
Undifferentiated cancer	30 (3.2%)	33 (10.4%)	< 0.001	3.223 (1.900–5.467)	< 0.001
TNM stage (≥III)	429 (45.1%)	137 (53.1%)	0.529	NA	

CEA, carcinoembryonic antigen; CI, confidence interval; CRC, colorectal cancer; dNLR, derived neutrophil-to-lymphocyte ratio; DM, diabetes mellitus; EGFR, epidermal growth factor receptor; FHX, family history; LCC, left-sided colorectal cancer; OR, odds ratio; RCC, right-sided colorectal cancer; TNM stage, tumor-node-metastasis staging system; WBC, white blood cell.

**Table 3:** Multivariate analysis of sex-specific different characteristics in colon cancer.

Sex	Variable	Location	OR (95% CI)	*p*
Men	Anemia	LCC	1	<0.001
		RCC	2.620 (1.841–3.727)	
	Undifferentiated cancer	LCC	1	0.077
		RCC	1.968 (0.929–4.169)	
Women	Age ≥ 65	LCC	1	0.031
		RCC	1.587 (1.044–2.414)	
	Anemia	LCC	1	0.001
		RCC	1.976 (1.301–3.000)	
	Abnormal WBC count	LCC	1	0.014
		RCC	0.587 (0.383–0.900)	
	Undifferentiated cancer	LCC	1	<0.001
		RCC	6.081 (2.660–13.905)	

CI, confidence interval; LCC, left-sided colorectal cancer; OR, odds ratio; RCC, right-sided colorectal cancer; WBC, white blood cell.

## Data Availability

The raw data supporting the conclusions of this study will be made available by the authors upon request.

## References

[1] Siegel R.L., Wagle N.S., Cercek A., Smith R.A., Jemal A. (2023). Colorectal cancer statistics, 2023. CA Cancer J. Clin..

[2] Baran B., Mert Ozupek N., Yerli Tetik N., Acar E., Bekcioglu O., Baskin Y. (2018). Difference Between Left-Sided and Right-Sided Colorectal Cancer: A Focused Review of Literature. Gastroenterol. Res..

[3] Helvaci K., Eraslan E., Yildiz F., Tufan G., Demirci U., Berna Oksuzoglu O., Yalcintas Arslan U. (2019). Comparison of clinicopathological and survival features of right and left colon cancers. J. Balk. Union. Oncol..

[4] Langman J., Sadler T.W. (1985). Langman’s Medical Embryology.

[5] Bufill J.A. (1990). Colorectal cancer: Evidence for distinct genetic categories based on proximal or distal tumor location. Ann. Intern. Med..

[6] Sideris M., Adams K., Moorhead J., Diaz-Cano S., Bjarnason I., Papagrigoriadis S. (2015). BRAF V600E mutation in colorectal cancer is associated with right-sided tumours and iron deficiency anaemia. Anticancer Res..

[7] Mik M., Berut M., Dziki L., Trzcinski R., Dziki A. (2017). Right- and left-sided colon cancer—clinical and pathological differences of the disease entity in one organ. Arch. Med. Sci..

[8] Kim K., Kim Y.W., Shim H., Kim B.R., Kwon H.Y. (2018). Differences in clinical features and oncologic outcomes between metastatic right and left colon cancer. J. Balk. Union. Oncol..

[9] Baraibar I., Ros J., Saoudi N., Salva F., Garcia A., Castells M.R., Tabernero J., Elez E. (2023). Sex and gender perspectives in colorectal cancer. ESMO Open.

[10] Liu K.A., Mager N.A. (2016). Women’s involvement in clinical trials: Historical perspective and future implications. Pharm. Pract..

[11] Abancens M., Bustos V., Harvey H., Mcbryan J., Harvey B.J. (2020). Sexual Dimorphism in Colon Cancer. Front. Oncol..

[12] Wood G., Grenader T., Nash S., Adams R., Kaplan R., Fisher D., Maughan T., Bridgewater J. (2017). Derived neutrophil to lymphocyte ratio as a prognostic factor in patients with advanced colorectal cancer according to RAS and BRAF status: A post-hoc analysis of the MRC COIN study. Anticancer Drugs.

[13] Sharma A., Athanasopoulos S., Li Y., Sanders S.N., Kumarasamy E., Campos L.M., Lakhwani G. (2022). Probing Through-Bond and Through-Space Interactions in Singlet Fission-Based Pentacene Dimers. J. Phys. Chem. Lett..

[14] Hu W., Yang Y., Li X., Huang M., Xu F., Ge W., Zhang S., Zheng S. (2018). Multi-omics Approach Reveals Distinct Differences in Left- and Right-Sided Colon Cancer. Mol. Cancer Res..

[15] Yang S.Y., Cho M.S., Kim N.K. (2018). Difference between right-sided and left-sided colorectal cancers: From embryology to molecular subtype. Expert Rev. Anticancer Ther..

[16] Goncalves C., Duarte L., Alves J.J.C. (2023). Differences Between Right and Left Colon Cancer in Beira Interior. Cureus.

[17] Kalantzis I., Nonni A., Pavlakis K., Delicha E.M., Miltiadou K., Kosmas C., Ziras N., Gkoumas K., Gakiopoulou H. (2020). Clinicopathological differences and correlations between right and left colon cancer. World J. Clin. Cases.

[18] Nitsche U., Stogbauer F., Spath C., Haller B., Wilhelm D., Friess H., Bader F.G. (2016). Right Sided Colon Cancer as a Distinct Histopathological Subtype with Reduced Prognosis. Dig. Surg..

[19] Qaderi S.M., Galjart B., Verhoef C., Slooter G.D., Koopman M., Verhoeven R.H.A., De Wilt J.H.W., Van Erning F.N. (2021). Disease recurrence after colorectal cancer surgery in the modern era: A population-based study. Int. J. Color. Dis..

[20] Vayrynen J.P., Tuomisto A., Vayrynen S.A., Klintrup K., Karhu T., Makela J., Herzig K.H., Karttunen T.J., Makinen M.J. (2018). Preoperative anemia in colorectal cancer: Relationships with tumor characteristics, systemic inflammation, and survival. Sci. Rep..

[21] Almilaji O., Parry S.D., Docherty S., Snook J. (2021). Evidence for improved prognosis of colorectal cancer diagnosed following the detection of iron deficiency anaemia. Sci. Rep..

[22] Almilaji O., Parry S.D., Docherty S., Snook J. (2022). Colorectal cancer and the blood loss paradox. Frontline Gastroenterol..

[23] Estevao D., Da Cruz-Ribeiro M., Cardoso A.P., Costa A.M., Oliveira M.J., Duarte T.L., Da Cruz T.B. (2023). Iron metabolism in colorectal cancer: A balancing act. Cell Oncol..

[24] Bai J., Chen H., Bai X. (2021). Relationship between microsatellite status and immune microenvironment of colorectal cancer and its application to diagnosis and treatment. J. Clin. Lab. Anal..

[25] Kang S., Na Y., Joung S.Y., Lee S.I., Oh S.C., Min B.W. (2018). The significance of microsatellite instability in colorectal cancer after controlling for clinicopathological factors. Medicine.

[26] Xiao H., Yoon Y.S., Hong S.M., Roh S.A., Cho D.H., Yu C.S., Kim J.C. (2013). Poorly differentiated colorectal cancers: Correlation of microsatellite instability with clinicopathologic features and survival. Am. J. Clin. Pathol..

[27] Bellio H., Fumet J.D., Ghiringhelli F. (2021). Targeting BRAF and RAS in Colorectal Cancer. Cancers.

[28] Kim N. (2021). Sex-and gender-related issues of gut microbiota in gastrointestinal tract diseases. Korean J. Gastroenterol..

[29] Flores R., Shi J., Fuhrman B., Xu X., Veenstra T.D., Gail M.H., Gajer P., Ravel J., Goedert J.J. (2012). Fecal microbial determinants of fecal and systemic estrogens and estrogen metabolites: A cross-sectional study. J. Transl. Med..

[30] Rebersek M. (2021). Gut microbiome and its role in colorectal cancer. BMC Cancer.

[31] Shivers K.Y., Amador N., Abrams L., Hunter D., Jenab S., Quinones-Jenab V. (2015). Estrogen alters baseline and inflammatory-induced cytokine levels independent from hypothalamic-pituitary-adrenal axis activity. Cytokine.

[32] Chervenak J. (2009). Bioidentical hormones for maturing women. Maturitas.

[33] Torrens J.I., Sutton-Tyrrell K., Zhao X., Matthews K., Brockwell S., Sowers M., Santoro N. (2009). Relative androgen excess during the menopausal transition predicts incident metabolic syndrome in midlife women: Study of Women’s Health Across the Nation. Menopause.

[34] Refaat B., Aslam A., Idris S., Almalki A.H., Alkhaldi M.Y., Asiri H.A., Almaimani R.A., Mujalli A., Minshawi F., Alamri S.A. (2023). Profiling estrogen, progesterone, and androgen receptors in colorectal cancer in relation to gender, menopausal status, clinical stage, and tumour sidedness. Front. Endocrinol..

[35] White A., Ironmonger L., Steele R.J.C., Ormiston-Smith N., Crawford C., Seims A. (2018). A review of sex-related differences in colorectal cancer incidence, screening uptake, routes to diagnosis, cancer stage and survival in the UK. BMC Cancer.

[36] Yang Y., Wang G., He J., Ren S., Wu F., Zhang J., Wang F. (2017). Gender differences in colorectal cancer survival: A meta-analysis. Int. J. Cancer.

